# Targeting Tumor Endothelial Cells with Nanoparticles

**DOI:** 10.3390/ijms20235819

**Published:** 2019-11-20

**Authors:** Yu Sakurai, Hidetaka Akita, Hideyoshi Harashima

**Affiliations:** 1 Graduate School of Pharmaceutical Sciences, Chiba University, Chiba 260-8675, Chiba, Japan; akitahide@chita-u.jp; 2 Faculty of Pharmaceutical Sciences, Hokkaido University, Sapporo 060-0812, Hokkaido, Japan; harasima@pharm.hokudai.ac.jp

**Keywords:** nanoparticles, drug delivery system, tumor endothelial cells, anti-angiogenic therapy, nucleic acids

## Abstract

Because angiogenesis is a major contributor to cancer progression and metastasis, it is an attractive target for cancer therapy. Although a diverse number of small compounds for anti-angiogenic therapy have been developed, severe adverse effects commonly occur, since small compounds can affect not only tumor endothelial cells (TECs), but also normal endothelial cells. This low selectivity for TECs has motivated researchers to develop alternate types of drug delivery systems (DDSs). In this review, we summarize the current state of knowledge concerning the delivery of nano DDSs to TECs. Their payloads range from small compounds to nucleic acids. Perspectives regarding new therapeutic targets are also mentioned.

## 1. Introduction

Angiogenesis is a crucial step in cancer progression. In 1971, Folkman proposed an anti-angiogenic therapy hypothesis in which the inhibition of angiogenesis would lead to tumor shrinkage because the growth of tumor tissue is necessarily accompanied by angiogenesis [1]. Since this report, a large number of studies dealing with anti-angiogenic therapy have been reported. Basic fibroblast growth factor (bFGF) was first discovered as an enhancer of angiogenesis [2]. Vascular endothelial cell growth factor (VEGF) was subsequently identified and is now thought to be a dominant factor in tumor angiogenesis [3,4]. Based on the discovery of VEGF, bevacizumab, a mouse antibody against human VEGF, was approved by the food and drug administration (FDA) in 2004 and is currently used in the treatment of various types of cancers worldwide. However, these angiogenic factors also play a pivotal role in the physiological state. Anti-angiogenic therapy is sometimes accompanied by severe adverse effects, including bowel perforation and hemorrhage [5]. The specific delivery of therapeutics to the tumor vasculature, and not to the normal vasculature, would be a potent strategy.

Therefore, tumor vasculature targeting has become an interesting target for researchers in the field of nano drug delivery systems (DDS). This is because nano DDSs can be designed for controlled release and targeting an on-target organ using functional devices [6]. These features permit adverse effects to be overcome by circumventing accumulation in off-target organs. In addition, nucleic acids medicine, including small interfering RNA (siRNA) and micro RNA (miRNA), have received considerable attention because these therapeutics are capable of regulating target genes in a sequence dependent manner. RNA interference was discovered by Fire and Mello in 1998 based on the result that the incorporation of double stranded RNA to *C. elegans* induced gene silencing with a complementary sequence [7]. In 2001, Elbashir reported that short RNA composed of about 21 mers could inhibit target genes, even in mammalian cells, without any obvious toxicities [8]. Since this discovery, much effort has been made to develop systems for delivering nucleic acids. This is because it has become easier to design a sequence of nucleic acids of interest since the development of DNA and RNA sequencing. Since nucleic acids are rapidly degraded in the body, it is necessary to develop DDSs to carry them. As of this writing, a huge number of studies on nucleic acids delivery have been reported [9]. We herein also summarized nucleic acids-based medicine to tumor endothelial cells (TECs).

Cancer cells are the primary target for DDSs, since DDSs with a prolonged circulation time were reported to passively accumulate in tumor tissue in a tumor-bearing mouse model, a process that is called “the enhanced permeability and retention” (EPR) effect [10]. A number of studies have been carried out in this area [11]. However, a recent study revealed that EPR effect-based nanomedicines showed weaker effects in the clinic than in animal models [12]. One cause of this difference between a clinical trial and a non-clinical model would be attributed to the dense stroma in tumor tissue that inhibit the penetration of nanoparticles. In other words, it appears to be difficult for nano-sized therapeutics to approach cancer cells through their dense stromal. On the contrary, TECs would be easy to access since they face the blood stream. Taken together, TECs would be promising targets from the view point of DDSs. In this review, we summarize the latest updates associated with nano DDSs targeting TECs.

## 2. For Targeting Tumor Endothelial Cells

As mentioned above, a targeting ligand would be required for delivering a nano DDS to TECs. It would be important to reveal the difference between TECs and normal endothelial cells for achieving selective targeting. In fact, TECs have a chromosomal aberration similar to tumor cells [13]. Several markers, such as suprabasin [14] and lysyl oxidase [15], have now been now identified. These characteristic protein expressions in TECs would be a promising target due to their specificity. Details of the importance in cancer progression and dissemination should be covered in other reviews in this issue. As we previously reviewed the vasculature-targeting nanoparticles [16,17], there are numerous ligands that can be used for targeting TEC-specific proteins, such as peptides, sugars, nucleic acids aptamers, and cationic charged materials for the features of TECs. These compounds are discussed below.

### 2.1. Peptide

#### 2.1.1. RGD Motif

RGD (Arginine-Glycine-Aspartic acid), a popular motif, can specifically bind TECs and some types of cancer cells. The RGD motif recognizes integrin heterodimers between the αV unit (CD51) and the β3 unit (CD61) on the surface of TECs, which is a receptor for vitronectin and is involved in angiogenesis [18,19]. The cyclized RGD peptide (cRGD) is widely used due to its high affinity for integrin through a constrained structure (Table 1). Amin et al., using intravital imaging, observed that cRGD-modified liposomes (LPs) were localized both in vessels and in the perivascular region by [20]. They compared several RGD peptides (cRGDfK, RGDyC and RGDf[N-methyl]C). Of these, RGDf[N-methyl]C was found to be superior to other peptides on terms of the ability to enhance the cellular uptake of LPs. Therefore, they concluded that hydrophobicity would be important for the internalization of the cRGD peptide. Various molecules have been developed for targeting TECs in addition to LPs. Dendrimers, an oligomer with a branched structure, are also used as drug carriers, [21]. Li et al. decorated the polyamidoamine (PAMAM) dendrimer with cRGD [22]. Using this, they demonstrated that the fluorescence-labeled cRGD-PAMAM dendrimer accumulated in tumor tissue a three-fold higher level than the non-ligand PAMAM dendrimer. They showed that cRGD-PAMAM delivered fluorescence for orthotopic glioma through the blood-brain tumor barrier (BBTB). Thus, cRGD modification allows various types of nano DDSs to accumulate in tumor vessels. A cargo of nano DDS is not limited to small molecules. Sun et al. encapsulated paclitaxel (PTX) and the KLA peptide, which disrupted the mitochondrial membrane, and consequently induced apoptosis, into LPs [23,24]. Systemically injected PTX and KLA peptide-co-loaded LPs inhibited tumor growth in a murine breast cancer model [25]. They then showed that the CD31-positive area was decreased when their system was injected. Natural compounds that have anti-angiogenic effects, such as curcumine, are also preferable cargoes [26]. Nik et al. encapsulated Galbanic acid, which is a sesquiterpene coumarin extracted from Ferula, into cRGD-modified LPs [27]. They demonstrated that LPs encapsulating Galbanic acid decreased angiogenic vessels in a chicken chorioallantoic membrane angiogenesis (CAM) model. Lu et al. reported on the preparation of a cRGD-decorated PAMAM dendrimer encapsulating arsenic trioxide [28], which is known to be a conventional Chinese medicine for treating cancer. The PAMAM dendrimer encapsulating arsenic trioxide arrested the cell cycle in the G2/M phase and exhibited restricted proliferation in glioma cells. The use of a combination of this DDS with radiotherapy has been also studied. Boron neutron capture therapy (BNCT) is one such radiotherapy modality. In BNCT, cancer cells are killed by fission reactions of alpha rays via thermal nuclear capture by a stable isotope ^10^B [29]. As the length of alpha ray fissions is very short (< 10 μm), BNCT is highly selective to the ^10^B-taken up cells. Therefore, targeting is essential for BNCT in order to avoid accumulating in off-target cells and consequent adverse effects. Kang et al. reported that sodium borocaptate-loaded LPs can efficiently kill HUEVC and U87 cells by exposure to thermal neutrons [30].

In angiogenesis, not only proliferating new vessel, but also the vasculogenic mimicry (VM) of tumor cells contribute to the increased level of micro vessels. The term VM means that cancer cells mimic endothelial cells, which results in the formation of vessel-like structures that supply growth factors and oxygen [35]. Wang et al. [31] prepared cRGD-conjugated heparin, which acts as a therapeutic against VM and conjugation of the ligand backbone. They demonstrated that the cRGD-conjugated heparin efficiently inhibited tube formation in an in vitro study and inhibited tumor growth in ovarian cancer-bearing mice by suppressing VM formation.

We developed siRNA-loaded lipid nanoparticles (LNP), a multi-functional envelope-type nano-device (MEND) using the pH-responsive cationic lipid, YSK05 and succeeded in delivering siRNA to cancer cells in a renal cell carcinoma model [36,37]. Recently, in order to target TECs, we modified an MEND with cRGD (RGD-MEND) by post-modification of the conjugates of cRGD-PEG-lipid [32]. In an in vitro study, the RGD-MEND specifically delivered siRNA to integrin positive human umbilical vein endothelial cells (HUVEC), but not to HEK293T cells. We also revealed that the RGD-MEND accumulated in TECs in tumor-bearing mice, and that an RGD-MEND encapsulating anti-VEGFR2 siRNA significantly inhibited tumor growth (50% effective dose: 0.75 mg/kg). The RGD-MEND could also be applied to a lung metastasis model [33]. In the case of a lung-metastasis model, VEGFR2 inhibition by the RGD-MEND failed to inhibit tumor growth. We then changed the target gene from VEGFR2 to the delta-like ligand4 (DLL4). A previous study reported that DLL4 inhibition by an antibody (Ab) inhibited “non-productive” vessels, and consequently induced the shrinkage of the tumor mass [38]. The continuous injection of the RGD-MEND encapsulating siRNA against DLL4 substantially prolonged overall survival of lung-metastasis model mice. We also attempted to establish a new therapy via regulating the tumor microenvironment by delivering siRNA to TECs. It was revealed that the continuous inhibition of VEGFR2 on TECs improved the poor intratumoral distribution of nanoparticles [39]. This improvement was also dependent on the type of cancer. VEGFR2 inhibition was effective in vessel-rich cancer types (renal cell carcinoma, ovarian cancer, hepatocellular carcinoma), but not in vessel-poor cancer types (colorectal cancer, pancreatic cancer) [40]. Our data suggest that TECs would be a key regulator of the tumor microenvironment.

iRGD (CRGDKRGPDEC) is a newly identified peptide produced by in vivo T7 phage display with a similar sequence to conventional cyclic RGD [41]. iRGD not only binds to the tumor vasculature but also deeply penetrates the tumor mass. This feature is very important because nano DDSs penetrate tumor tissues with difficulty due to the dense stroma, as mentioned above. The penetration of iRGD occurs in three steps; the RGD motif binds to αv integrins on TECs, and is subsequently cleaved proteolytically which then exposes a binding motif for neuropilin-1, which finally mediates penetration into the tumor mass. That explains why iRGD-modified nano DDSs target both cancer cells and TECs. Li et al. developed iRGD-conjugated mesoporous silica nanoparticles (MSN), to which hydrophobic drugs could be absorbed. They loaded both an anti-angiogenic agent (combretastatin A4, CA4) and a chemotherapeutic agent (DOX) [34]. MSN loading moth drugs demonstrated an effective inhibition of tumor growth with cervical cancer-bearing mice via the disruption of the tumor vasculature and cancer cell apoptosis. Song et al. reported that iRGD-modified LPs encapsulating DOX inhibited angiogenesis in a breast cancer model [42].

#### 2.1.2. NGR

The NGR motif is also famous for recognizing TECs. Aminopeptidase N (APN, CD13) is responsible for the binding of the NGR peptide to the tumor vasculature. The NGR peptide was discovered by an in vivo phage display against a human breast cancer xenograft [43]. The NGR peptide is also widely used for targeting the tumor vasculature as summarized in Table 2 [44]. Huang et al. developed NGR-modified LPs that contain the anti-angiogenic agent CA4 [45]. They showed that NGR-modified LPs recognized the VM of glioma and efficiently delivered drugs to glioma xenografts. Hu et al. revealed that DOX-loaded MSN modified with the NGR peptide could be delivered to the tumor vasculature in BBTB [46]. The NGR peptide is often combined with other ligands to increase the binding affinity to TECs. Yang et al. modified LPs with both the NGR peptide and a cell penetrating peptide (CPP), which is a generic term for cationic peptides that significantly enhance cellular uptake [47,48]. They showed that dual-ligand modification synergistically improved the targeting ability of these systems.

The iNGR peptide was recently designed based on the iRGD peptide [51]. As mentioned above, iRGD was proteolytically cleaved after binding to integrin, and consequently the CendR motif (R/KXXR/K) appeared. This CendR motif was then recognized by neuropilin-1. In the iNGR sequence, the NGR sequence was incorporated into this CendR motif instead of the RGD sequence. Based on this discovery, Kang et al. developed an iNGR-modified Poly(lactic-co-glycolic) acid (PLGA) [49], which was approved by the FDA as a drug carrier due to its high biocompatibility. In this article, the intravenous injection of PTX-loaded iNGR-PLGA decreased the tumor vasculature and consequently prolonged overall survival in glioma-bearing mice model. Similarly, Zhou et al. reported that the iNGR-modified DOX-LP exhibited a significant anti-tumor effect in an orthotopic glioma-model [50]

#### 2.1.3. Other Peptides

The examples for TEC-targeting DDS using other peptides were summarized in Table 3. The CGKRK peptide, which was identified by a phage display, binds to both angiogenic endothelial cells and tumor cells with a high affinity [52]. The target molecule of CGKRK is known to be heparan sulfate, which is located on the surface of TECs. Hu et al. developed a CGKRK peptide with an affinity to poly(caprolactone) (PCL) for anti-angiogenic therapy [53]. They showed that the CGKRK-modified PCL was co-localized on TECs, and that the delivery of PTX with this nano DDSs efficiently inhibited glioma growth. Lu et al. combined the CGKRK peptide with Pep-1, which binds to the Interleukin 13 receptor α2 (IL-13Rα2), which is highly expressed in glioma cells [54]. They modified PTX-loaded PLGA nanoparticles with these two peptides (PC-NP-PTX) for the dual-targeting to both cancer cells and TECs. The intravenous injection of PC-NP-PTX resulted in a significantly improved overall survival. In vitro studies revealed that PC-NP-PTX more efficiently inhibited the migration of HUVEC by transwell analysis than modification with the single peptide PLGA. Therefore, they concluded that the dual-modification of peptides was efficient for anti-angiogenic therapy. A cyclic 9-mer peptide, GX1 (CGNSNPKSC), which was also screened by means of an in vivo phage display, showed a high affinity for the gastric cancer vasculature [55]. Zhang et al. modified chitosan with the GE1 peptide [56]. Chitosan, whose structure is β-(1→4)-linked D-glucosamine and N-acetyl-D-glucosamine units is prepared by the deacetylation of chitin, and is used for drug delivery, regenerative medicine, wound healing and related processes [57]. In this articles, DTX-loaded chitosan modified with the GE1 peptide inhibited tumor growth in a gastric cancer model.

Some nano DDSs have been developed based on the fact that angiogenic vessels are in an inflammatory condition. Inflammation induces the overexpression of VEGFRs, cell adhesion molecules such as E-selectin and the intercellular adhesion molecule (ICAM). F56 (WHSDMEWWYLLG) is a peptide that was isolated by a phage display peptide library, which strongly binds to the highly expressed VEGFR-1 on TECs [62]. Sharnay et al. modified the N-(2-hydroxypropyl) methacrylamide (HPMA) copolymer with F56 to produce an F56-modified HPMA [58]. The F56-modified HPMA polymer could strongly bind to bEnd.3 cells, but not cancer cells (B16F10, 3LL, HT-29). F56-modified DOX-loaded HPMA significantly prolonged the overall survival of mice that had been inoculated with murine lung cancer and melanoma cells. Luan et al. demonstrated that depleting excessive vessels by PTX resulted in a normalized tumor microenvironment [59]. After the injection of F56-modified poly (lactic acid) (PLA) encapsulating PTX, they found that thrombospondin-1 (TSP-1), a major negative regulator of angiogenesis [63], secreted by TECs was upregulated in addition to a decrease in angiogenic vessels. Han et al. used the E-selectin binding peptide (Esbp; CDKDKDITWDQLWDLMK) for targeting TECs. E-selectin is an inducible transmembrane protein that is selectively expressed on activated endothelial cells in response to cytokines, such as TNF-α and IL1β, during inflammation and cancer progression [64]. They established hyaluronic acid conjugated to PTX micelles. The systemic administration of this micelle inhibited breast cancer metastasis to lung tissue by decreasing the density of angiogenic vessels in the primary tumor.

Fukuta et al. proposed a unique strategy aimed at targeting endothelial progenitor cells (EPCs) [61]. EPCs are typically defined as a cell population that can differentiate into endothelial cells and contribute to the formation of new blood vessels [65]. They first identified the peptide (ASSHNGC) by in vitro biopanning against human EPC with M13 phage. They confirmed that DOX-LP particles modified with this peptide were co-localized on TECs after their systemic injection. Finally, EPC-targeting DOX-LP exerted a robust therapeutic effect in a colon cancer model.

### 2.2. Other Molecules

#### 2.2.1. Antibodies

The antibody (Ab) and its fragmented derivatives are popular ligands that allowing nano DDSs to target cells of interest [66]. Whole IgG, (Fab)_2_ and Fab’ are popularly used for targeting TECs. Several studies on Ab-targeted nano DDSs toward TECs have appeared as shown in Table 4. VEGFR2 is a potent target receptor for Ab-conjugated nano DDSs. Li et al. developed anti-mouse VEGFR2 immunoglobulin (IgG) conjugated for hemangioma therapy [67]. IgG was linked to LPs by a 1,4-addition reaction between a thiol group of IgG and a maleimide group on the surface of the LP. The authors encapsulated sodium morrhuate, which was is a mixture of sodium salts of the saturated and unsaturated fatty acids present in cod-liver oil. This reagent disrupted endothelial cells and promoted blood coagulation and thrombosis [68]. They showed that the Ab-conjugated LP induced apoptosis in hemangioma derived-TECs. The whole IgG molecule is not the only form that can be used in conjugation reactions. Orleth et al. first enzymatically fragmented IgG against VEGFR2 and 3 to Fab’, and then conjugated it to a DOX-loaded LP via covalent linkage [69]. They next examined the density of micro vessels in tumor tissue using RIP1-Tag2 transgenic mice. As a result, two Ab-conjugated LPs exhibited a more reduction than single Ab-conjugated LP. Zhou et al. used Ab against CD105 (Endoglin) [70]. They prepared a microbubble LP that encapsulated otafluoropropane (C_3_F_8_). Encapsulated C_3_F_8_ was very quickly vaporized in response to exogenous ultrasound energy, and consequently, induced pore formation in cellular membranes (so-called sonoporation). Sonoporation is a technology that results in the efficient release of its payload [71]. Plasmid DNA (pDNA) encoding endostatin, which inhibits angiogenesis by binding and inhibiting the α5β1 integrin [72], was complexed with an anti-CD105 Ab-conjugated microbubble LP. The injection of this LP substantially inhibited tumor growth in a breast cancer model via an anti-angiogenic effect by endostatin produced from encapsulated pDNA [73]. Guo et al. modified an LP loaded with siRNA against lipocalin2 with an anti-ICAM-1 Ab [74]. Lipocalin 2, a member of the lipocalin protein superfamily, is a positive regulator of angiogenesis [75]. They showed that the siRNA-loaded LP inhibited angiogenesis by the siRNA-mediated silencing of lipocalin 2 using a CAM assay. There is a report focusing on the immune function of TECs. Cancer cells can evade immunosurveilance because of the abnormal circumstances caused by the tumor microenvironment [76]. Huang et al. studied the α1,3-galactosyltransferase (α1,3 GT) synthesizing carbohydrate epitope Galα1-3Galβ1-4GlcNAc-R on the cell surface, which is a major barrier to xenotransplantation [77]. They aimed at transfecting TECs with pDNA encoding this enzyme and the consequent host rejection against TECs by IgM against this Galα1-3Galβ1-4GlcNAc-R epitope [78]. To deliver pDNA to TECs, a single chain variable fragment (scFv) against endoglin was used as a targeting ligand. The structure of scFv is fusion protein of Fv moiety from heavy chain and light chain of Ab. The systemic injection of scFv-modified LP encapsulating encoding α1,3 GT induced the production of IgM against the Galα1-3Galβ1-4GlcNAc-R epitope. The expression of Galα1-3Galβ1-4GlcNAc-R for TECs by scFv LP-pDNA encoding α1,3 GT resulted in the production of an IgM antibody and consequently TECs were rejected. This rejection significantly prolonged overall survival in a lung cancer-bearing model via anti-angiogenic effect.

#### 2.2.2. Cationic Components

LPs with cationic lipids are popular platforms for targeting the angiogenic vasculature (Table 5), since it was reported that cationic LPs could be preferably delivered to inflamed, angiogenic vessels both in the tumor tissue and chronically inflamed lungs [79]. Another report suggested that neutral and anionic LPs were not taken up by angiogenic vessels [80]. Although they concluded that angiogenic TECs would express high levels of anionic proteins on the cell surface, the exact mechanism responsible for the preferable accumulation of cationic LPs was not examined. One of the most potent examples is AtuPLEX (also referred to as Atu027). AtuPLEX is a lipoplex with siRNA, and is composed of β-L-arginyl-2,3-L-diaminopropionic acid-N-palmitoyl-N-oleyl-amide trihydrochloride (referred to as AtuFECT01) and a neutral lipid 1,2-diphytanoyl-sn-glycero-3-phosphoethanolamine [81,82]. These preparations were found to successfully inhibit lung metastasis by the suppression of protein kinase N3 (PKN3) via an anti-angiogenic effect [83,84]. The use of AtuPLEX in Phase I clinical study is currently underway for patients with advanced solid tumors [85,86]. Thus, a cationic charge is a potentially hopeful strategy for anti-angiogenic therapy. Luo et al. recently reported on a therapeutic effect caused by cationic LPs with porphyrin-phospholipid (PoP) conjugates encapsulating DOX. PoP oxidizes cationic lipids dioleoyl-3-trimethylammonium propane (DOTAP) in response to near-infrared (NIR) light, and consequently, the encapsulated DOX is released from the LPs [82]. When human pancreatic cancer-bearing mice were treated with this LPs and NIR light (100 J/cm^2^), tumor growth was significantly inhibited via an anti-angiogenic effect.

#### 2.2.3. Saccharide, Aptamer and Others

Sialyl LewisX (SLX) is a tetrasaccharide composed of N-acetylneuraminic acid, two galactose units and an N-acetylglucosamine unit. SLX was first discovered as a cancer antigen, which recognizes E-selectin on the surface of endothelial cells, specifically under inflammatory conditions [87]. Alekseeva et al. reported that SLX-modified LP was internalized in TNF-α-activated HUVEC but not in non-activated-HUVEC [88]. Although SLX is a promising ligand for use in inflammatory TECs, perhaps due to fact that SLX is both expensive and rare, there are few reports on in vivo studies [16].

Nucleic acids aptamers are composed of single stranded DNA and/or RNA with sequence-dependent secondary structures [89]. An aptamer that selectively binds to target molecules was identified by the systematic evolution of ligands by the exponential Enrichment (SELEX) approach, in which oligonucleotide libraries with random sequences are subjected to repeated selection to target molecules/cells and the bound sequences are subsequently amplified by PCR [90]. Finally, a few oligonucleotides are identified that are able to strongly bind to targets by sequencing after several selection steps. We previously identified an aptamer which can specifically bind to TECs, AraHH001 by cell-based SELEX [91]. Moreover, we revealed that LPs modified with the AraHH001 aptamer binds to TECs in a renal cell carcinoma model [92].

### 2.3. Beyond Tumor Blood Endothelial Cell–Lymphatic System

As described above, nano DDSs for TECs are reaching a mature state. It will be important for researchers in the field of DDS to explore a next target. We currently conclude that the lymphatic system would be “blue ocean”. The lymphatic system is the third vascular system, and plays a key role in the drainage of interstitial fluid. Lymphatic endothelial cells (LECs), which lymphatic vessel consists of, have been subjects of much attention. This would be because lymph systems are more difficult to be observed, and hence, be biologically examined. Currently, markers of LECs include molecules such as podoplanin, the lymphatic vessel endothelial hyaluronan receptor-1 (LYVE-1) and the Prospero homeobox protein 1 (PROX1) [93]. The results of recent studies suggest that LECs are not simply a wall of a lymph aisle, but rather are a major regulator of immune systems. Lane et al. reported that IFN-γ in the tumor microenvironment upregulated the expression of the programmed death ligand 1 (PD-L1), and consequently attenuated immunotherapy by T cells [94]. Another report indicated that LYVE-1 on LECs are essential for the movement of dendritic cells to lymph nodes [95]. Thus, it appears that LEC play a key role in the immune system. Additionally, the majority of epithelial cancers metastasize by spreading via lymphatic vessels [96]. Despite this fact, the focus of most current studies is on blood metastasis. In 2001, some groups independently reported on a new mechanism of tumor lymphatic metastasis by the induction of lymphangiogenesis, which meant that LECs proliferate in response to the production of cytokines/chemokines, in tumor tissue [97]. Thus, LECs are likely to be a promising target for cancer therapy.

As of this writing, only a few reports on nano DDSs toward LECs have appeared. LyP-1 is one of the limited targeting peptides for LECs. LyP-1 was identified by an in vivo phage display against a MDA-MB-435 xenograft [98]. Yan et al. developed DOX-loaded LPs modified with the LyP-1 peptide. They showed that the LyP-1-modied LPs disrupted lymph vessels in tumor tissue by delivering DOX to LECs, and that the disruption inhibited lymph node metastasis [99]. Another group demonstrated that LyP-1 peptide-conjugated PLGA nanoparticles can be accumulated in lymph nodes with metastasis [100]. In the near future, much attention should be directed to LEC-targeting nano DDSs.

## 3. Conclusions

The tumor endothelial cell (TEC) is an attractive target, not only for biologists, but also for researchers in the field of drug delivery systems (DDSs). A number of nano DDSs targeting TEC systems have been developed, and some are currently used in clinical studies. We hope that TEC-targeting nano DDSs will be used for patients who are suffering from advanced cancers in the near future.

## Figures and Tables

**Table 1:** RGD-modified nanoparticles.

Name	Ligand	Carrier	Therapeutics	Cancer Type	Ref
SSLD	cRGDfK and cRGDyC	LP	DOX	colon and melanoma	[20]
cRGD-PAMAM	cRGDyC peptide	PAMAM dendrimer	fluorescence	breast cancer	[22]
RGD-KLA/PTX-Lips	cRGDyK	LP	PTX, KLA peptide	breast cancer	[23]
PLGba	cRGDyK	LP	Galbanic acid	colon cancer	[25]
cRGD-PAMAM	cRGDyC	PAMAM dendrimer	arsenic trioxide	orthotopic glioma	[28]
c(RGDyC)-LP	cRGDyK	LP	sodium borocaptate	glioma	[30]
RGD-heparin	cRGD	self-assembled heparin	heparin	ovarian cancer (vasculogenic mimicry)	[31]
RGD-MEND	cRGDfK	lipid nanoparticle	siRNA against VEGFR2	renal cell carcinoma, lung metastasis model	[32,33]
iRGD-MSN	iRGD	MSN	DOX, CA4	cervical cancer	[34]

**Table 2:** NGR-modified nanoparticles.

Name	Ligand	Carrier	Therapeutics	Cancer Type	Ref
NGR-SSL-CA4	NGR peptide	LP	CA4	glioma	[45]
MSN-DOX-PDA-NGR	NGR peptide	MSN	DOX	glioma	[46]
pcCPP/NGR-LP	NGR, CPP peptides	LP	siRNA against c-Myc	fibrosarcoma	[47]
iNGR-PLGA	iNGR peptide	PLGA	PTX	glioma	[49]
iNGR-SSL	iNGR peptide	LP	DOX	glioma	[50]

**Table 3:** Peptide-modified nanoparticles.

Name	Ligand	Carrier	Therapeutics	Cancer Type	Ref
CGKRK-NP	CGKRK	PCL	PTX	glioma	[53]
PC-NP-PTX	CGKRK, Pep-1 (IL-13Rα2)	PLGA	PTX	glioma	[54]
GX1-DGC-DCT	GX1	chitosan	DCT	gastric cancer	[56]
P-(F56)-DOX	F56	HPMA	DOX	melanoma, lung cancer, colon cancer	[58]
F56-PTX-NP	F56	PLA	PTX	breast cancer	[59]
Esbp-HA-PTX	Esbp	micelle	PTX	breast cancer	[60]
ASSHN-Lip	ASSHNGC	LP	DOX	melanoma, colon	[61]

**Table 4:** Antibody-modified nanoparticles.

Name	Ligand	Carrier	Therapeutics	Cancer Type	Ref
liposomal sodium morrhuate	IgG against VEGFR2	LP	sodium morrhuate	hemangioma	[67]
PLD	Fab’ against VEGFR2	LP	DOX	pancreatic cancer	[69]
CMB105	IgG against CD105	microbubble LP	pDNA encoding endostatin	breast cancer	[70,73]
ICAM-Lcn2-LP	IgG against ICAM-1	LP	siRNA against lipocalin 2	breast cancer	[74]
ENG-scFv-iLPs	scFv against endoglin	LP	pDNA encoding porcine α1,3 galactosyltransferase	lung cancer	[78]

**Table 5:** Cation-mediated nanoparticles.

Name	Ligand	Carrier	Therapeutics	Cancer Type	Ref
AtuPLEX	AtuFECT01	lipoplex	siRNA against PKN2	lung-metastasis model	[81,84]
DOTAP-PoP	DOTAP	LP	DOX released by NIR-light	pancreatic cancer	[82]

## Abbreviations

Ab	antibody
AtuFECT	β-L-arginyl-2,3-L-diaminopropionic acid-N-palmitoyl-N-oleyl-amide trihydrochloride
CA4	combretastatin A4
CAM	chicken chorioallantoic membrane angiogenesis
CPP	cell penetrating peptide
DDS	drug delivery system
DNA	deoxyribonucleic acid
DOTAP	dioleoyl-3-trimethylammonium propane
DOX	doxorubicin
EPR	enhanced permeability and retention
FDA	food and drug administration
HPMA	N-(2-hydroxypropyl)methacrylamide
HUVEC	Human umbilical vein endothelial cells
ICAM	intercellular adhesion molecule
IFN	interferon
IL	Interleukin
IgG	immunoglobulin
LNP	lipid nanoparticle
LP	liposome
LYVE-1	lymphatic vessel endothelial hyaluronan receptor-1
MEND	multi-functional envelope-type nano-device
MSN	mesoporous silica nanoparticle
NGR	Asn-Gly-Arg peptide
PAMAM	polyamidoamine
PCL	poly(caprolactone)
PKN3	protein kinase N3
PLA	poly(lactic acid)
PLGA	Poly(lactic-co-glycolic acid
PROX1	Prospero homeobox protein 1
PTX	Paclitaxel
PoP	porphyrin-phospholipid
RNA	ribonucleic acid
SELEX	systematic evolution of ligands by exponential Enrichment
SLX	Sialyl LewisX
TEC	Tumor endothelial cell
TNF	Tumor necrosis factor
VCAM	vascular cell adhesion molecule
VEGF	vascular endothelial cell growth factor
VEGFR	vascular endothelial cell growth factor receptor
VM	vascular mimicry
bFGF	basic fibroblast growth factor
cRGD	cyclized (Arg-Gly-Asp) peptide
miRNA	micro RNA
pDNA	plasmid DNA
scFv	single chain Fv
siRNA	small interfering RNA
α1,3 GT	α1,3-galactosyltransferase
